# Systems thinking as the integrative competency in undergraduate Family Medicine education: a cross-sectional evaluation of an integrated primary care block

**DOI:** 10.3389/fmed.2026.1870311

**Published:** 2026-07-31

**Authors:** Abdul Rehman Zia Zaidi, Baraa Alghalyini, Racha Kamal Khaled, Mohammad Ameen Alswes, Helen P. Batty

**Affiliations:** 1 Department of Family and Community Medicine, College of Medicine, Alfaisal University, Riyadh, Saudi Arabia; 2 School of Kinesiology and Health Science, York University, Toronto, ON, Canada; 3 Department of Family and Community Medicine, Temerty Faculty of Medicine, University of Toronto, Toronto, ON, Canada; 4 Department of Family Medicine, King Faisal Specialist Hospital and Research Center, Riyadh, Saudi Arabia

**Keywords:** clinical uncertainty, curriculum evaluation, Family Medicine, medical education, Primary Health Care, reflective practice, systems thinking, undergraduate medical education

## Abstract

**Background:**

Family Medicine and Primary Health Care anchor effective health systems, yet undergraduate curricula have given them limited dedicated space. The COM353 Family Medicine and Primary Care block at Alfaisal University was created to give these areas more dedicated curricular space, combining didactic teaching, Primary Health Care Center (PHCC) visits, structured reflection, and explicit engagement with clinical uncertainty.

**Methods:**

We conducted a single-institution, cross-sectional quantitative evaluation of third-year medical students who completed the block in January 2026. A 42-item Likert instrument, adapted from the Dundee Ready Education Environment Measure (DREEM) and the Groningen Reflection Ability Scale (GRAS) with block-specific items, captured perceptions across seven domains. Of 126 enrolled students, 96 responded (76.2%). Analyses included descriptive statistics, internal-consistency reliability, inter-domain correlations, and multiple linear regression. Reporting follows the STROBE guideline.

**Results:**

Domain means ranged from 3.90 to 4.27 (of 5), with all domains exceeding the scale midpoint by large margins (Cohen’s d, 0.92 to 1.58). Agreement rates ran from 63.7 to 80.7%. Internal consistency was excellent (Cronbach’s alpha = 0.988 overall; 0.940 to 0.974 across domains). Family Medicine and PHC Competencies achieved the highest mean (4.27) and reflective practice the lowest (3.90). On a single global rating, 87.5% of students rated the block positively (mean 3.80, SD 1.13). Multiple regression with six domains predicting perceived block value explained 78.2% of variance; *F*(6, 89) = 53.25, *p* < 0.001. Three domains independently predicted overall evaluation: Systems Thinking (beta = 0.727, *p* < 0.001), Reflection (beta = 0.295, *p* = 0.002), and Learning Environment (beta = 0.293, *p* = 0.013). Despite producing the highest absolute scores, Family Medicine Competencies did not independently predict overall evaluation, likely reflecting shared variance with Systems Thinking (r = 0.874).

**Conclusion:**

A purposefully integrated Family Medicine block joining community-based experience, reflective practice, and explicit work with uncertainty appears to produce favorable educational outcomes across multiple domains. Of these, perceiving healthcare as an interconnected system most strongly predicted students’ overall evaluation, suggesting that systems thinking acts as the integrative thread giving other curricular elements coherence.

## 1 Introduction

How should we train physicians for a profession in which certainty is the exception and routine clinical decisions must proceed on incomplete information? That question sits at the heart of medical education, and it is sharpest in primary care, the first-contact clinical service delivered in the community, and in the discipline of Family Medicine that provides it. Family physicians work as first-contact clinicians who handle undifferentiated presentations across every age group, disease process, and social circumstance. This clinical work is also the core of a wider system strategy. Global health bodies have championed Primary Health Care, the whole-of-society approach to organizing health systems, for decades, yet undergraduate curricula have not always followed by giving primary care and Family Medicine dedicated teaching space. Hospital wards and specialty rotations dominate clinical training; many students graduate having seen only fragments of the sustained, relationship-centered work that defines community practice (1).

Terminology in this field varies across health systems, and it helps to be precise from the outset. In the United Kingdom and Ireland the discipline is usually called general practice or primary care, whereas in Canada, the United States, and much of the Middle East, including Saudi Arabia, it is termed Family Medicine. The European Definition of General Practice/Family Medicine, maintained by WONCA Europe and used by the European Academy of Teachers in General Practice (EURACT) to shape training, treats general practice and Family Medicine as a single academic discipline with its own body of knowledge, organized around person-centered, continuous, comprehensive, and coordinated care (2, 3). Primary Health Care, by contrast, is the broader whole-of-society strategy for organizing health systems, first articulated at Alma-Ata and renewed in the 2018 Declaration of Astana, which combines three components: primary care and essential public health functions as the core of integrated services, multisectoral action on the determinants of health, and empowered people and communities (4–6). For clarity, we use Family Medicine to denote the discipline, Primary Health Care to denote the system-level approach, and primary care for the first-contact clinical service that forms one component of it. This distinction matters for what follows. The COM353 block is, before anything else, a primary care curriculum: it teaches the first-contact clinical work of Family Medicine. Strengthening that clinical capacity is also how undergraduate education contributes upstream to a Primary Health Care-oriented system, because competent generalist clinicians at the point of first contact are the element on which the wider strategy depends (6).

The downstream effects of this imbalance reach further than career choice. When primary care lives at the curricular margin, students absorb signals about what counts as serious medicine and where capable physicians belong. Parekh et al., working with reflective diaries kept by medical students at two UK institutions, traced precisely this pattern: a hidden curriculum that systematically undercut general practice through absent role models, by framing Family Medicine as a fallback, and by linking prestige to procedures (1). This pattern is not confined to any single country. Students worldwide move through training environments that privilege tertiary care, often without any deliberate counterweight describing what first-contact primary care actually involves, or why the generalist discipline of Family Medicine matters.

Evidence from the literature offers reasons for cautious optimism. A systematic review of Family Medicine clerkships found consistent gains in learning outcomes, clinical reasoning, and attitudes toward ambulatory practice following structured exposure (7). The same review, however, documented marked variability in duration, pedagogy, and assessment, making it hard to pinpoint which design features carry the greatest weight. More recently, Camarata et al. argued that medical education now operates within a VUCA environment, characterized by volatility, uncertainty, complexity, and ambiguity (8). The framing, borrowed from military strategy, captures the rate of change facing health professions training. In such conditions, curricula must build not only knowledge but adaptive competence, resilience, and comfort working in messy situations.

Among the competencies most needed in Primary Care is the capacity to tolerate, and work productively within, clinical uncertainty. Family Medicine is a particularly fertile setting in which to develop this capacity, because its early, undifferentiated presentations confront students with diagnostic uncertainty in a way that hospital specialty rotations, where many investigations are already complete, seldom do. Third-year medical students meet uncertainty constantly during clerkship transitions, but their responses vary widely. Kerr and Thompson followed how clerkship students communicated uncertainty with patients and showed that a willingness to acknowledge “I do not know” evolved over time and was shaped powerfully by the learning environment (9). That observation points to a pedagogical opening: if uncertainty tolerance can change, then curricula can be designed to grow it. Recent scholarship treats uncertainty tolerance as teachable and modifiable, supported through experiential exposure, psychologically safe environments, structured reflection, and direct engagement with ambiguous scenarios (10, 11).

Reflection has become a central mechanism linking clinical experience to professional growth. Drawing on Kolb’s experiential learning model, medical education increasingly embeds reflective activities not as assessment artifacts but as developmental processes that help students make sense of clinical encounters (12). Heydari and Beigzadeh described both barriers and facilitators to meaningful reflection, reporting that curriculum overload can hinder engagement, while students who reflect deeply describe richer integration of clinical and ethical perspectives (13). Qualitative work by Shikino et al. on community-oriented medical education reaches a similar conclusion: early experiential exposure paired with guided reflection promotes patient-centered orientation and a more grounded view of community health care (14). Reflection, then, cannot be added at the end of a placement; it must be woven through experiential learning from the start. These mechanisms are directly relevant here, because structured reflection is one of the four pedagogical pillars on which the COM353 block was built and one of the domains we set out to evaluate.

Despite these advances, quantitative studies that measure student perceptions across multiple educational domains within a single structured educational intervention in Primary Care remain uncommon. Most published evaluations focus on satisfaction or specialty interest, leaving open how integrated curricula shape self-assessed development in uncertainty tolerance, reflective capacity, systems thinking, and readiness for practice. Without that kind of evidence, educators struggle to make the case for the curricular time and resources Primary Care education needs.

The COM353 Family Medicine and Primary Care block at Alfaisal University was built specifically to address these gaps. Sited at a transitional point, where students hold foundational biomedical knowledge but limited sustained clinical exposure, the block bridges pre-clinical learning and future clerkships. It combines didactic teaching with immersive PHCC visits, structured reflective writing, and explicit engagement with what we call clinical courage: the willingness to engage thoughtfully with patients despite incomplete information. The aim is to recalibrate students’ picture of medicine before hospital-centric, specialty-focused norms harden.

The block is itself a product of academic collaboration operating at two levels: a university-to-university academic collaboration with the University of Toronto that informed the four-pillar curricular design, and a clinical-systems partnership with the Second Health Cluster in Riyadh that enabled student placements across multiple Primary Health Care Centers (the partnership architecture is described in detail in the Methods). This dual arrangement situates the block within the World Health Organization’s renewed Primary Health Care framework (5, 6) and connects directly to Sustainable Development Goal 3 (good health and well-being) and SDG 17 (partnerships for the goals).

To evaluate educational impact, we developed a 42-item survey instrument by adapting items from the Dundee Ready Education Environment Measure (DREEM), used widely to gauge perceptions of educational climate (15), and the Groningen Reflection Ability Scale (GRAS), a validated measure of reflective capacity (16). Block-specific items captured Family Medicine competencies, experiential learning, clinical uncertainty tolerance, systems thinking, and readiness for practice. The resulting instrument spans seven conceptual domains, giving a more complete picture of educational outcomes than narrower satisfaction surveys typically allow.

The present analysis quantifies how a structured, integrated educational intervention in Primary Care shapes medical students’ self-reported development across multiple competency domains. This is a quantitative study; learner perceptions were operationalized as numeric composite scores on a five-point Likert instrument and analyzed statistically. Two questions guided analysis. First, how favorably do third-year students rate seven educational domains, measured as mean composite scores on the Likert instrument, following the COM353 block? Second, which domains independently predict the perceived overall value of the block, tested by multiple linear regression? The findings speak to broader conversations about how to prepare students for contemporary practice, in which uncertainty is constant, systems thinking is essential, and reflective capacity has become a defining mark of excellence.

## 2 Materials and methods

### 2.1 Study design and reporting

We conducted a single-institution, cross-sectional quantitative evaluation of third-year medical students’ perceptions immediately following the COM353 Family Medicine and Primary Care block. The design was quantitative throughout: perceptions were captured as numeric Likert scores, aggregated into domain composites, and analyzed with descriptive and inferential statistics rather than through qualitative coding. Reporting follows the Strengthening the Reporting of Observational Studies in Epidemiology (STROBE) statement for cross-sectional studies; the completed STROBE checklist is provided as Supplementary File 1. Data collection was planned before block completion to capture perceptions while experiences were fresh. Outcomes are framed as learner perceptions rather than objective competency, an approach consistent with contemporary frameworks for curricular evaluation in health professions education.

### 2.2 Setting and curricular context

The study took place at the College of Medicine, Alfaisal University, Riyadh, Saudi Arabia. Alfaisal is a private, research-oriented institution offering a six-year undergraduate medical program organized around integrated organ-system blocks with progressive clinical exposure. COM353 is a mandatory block delivered to third-year students by the Department of Family and Community Medicine. Its placement is deliberate: by Year 3, students have built a foundation of biomedical knowledge but have had little sustained exposure to authentic clinical environments. The block functions as a bridge between pre-clinical learning and future clerkships, with the explicit goal of shaping students’ picture of medicine before hospital-centric norms set. For most students, COM353 is their first sustained, immersive encounter with primary care and the discipline of Family Medicine, rather than an abstract idea, and the first time the principles of Primary Health Care are made concrete. The block teaches primary care as clinical practice while situating it within the wider Primary Health Care approach: dedicated sessions on prevention and essential public health functions, the social determinants of health, health policy and regulation, and community-oriented care give students a working sense of the multisectoral and population dimensions that surround the individual consultation (5, 6).

**Figure 1:**
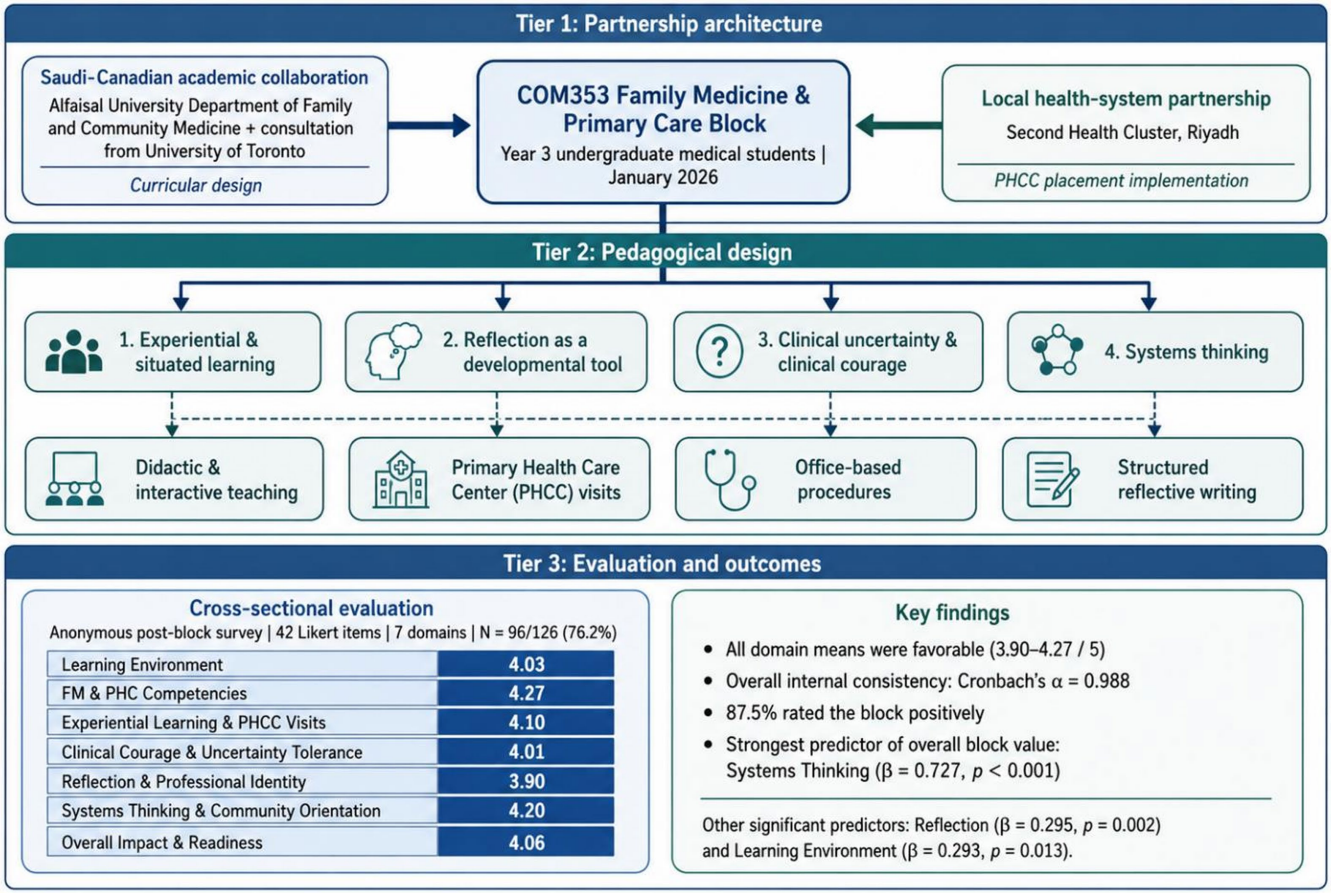
Conceptual design and key outcomes of the COM353 Family Medicine and Primary Care block. The diagram shows the block in three tiers. The top tier represents the partnership architecture: a Saudi-Canadian academic collaboration shaped the curricular design, with the Department of Family and Community Medicine at Alfaisal University leading the work in collaboration with the University of Toronto, while a separate clinical-systems partnership with the Second Health Cluster in Riyadh enabled placements across multiple Primary Health Care Centers. The middle tier shows the four pedagogical principles (experiential and situated learning, reflection as a developmental tool, explicit engagement with clinical uncertainty and clinical courage, and systems thinking) together with the educational components through which they were operationalized (didactic and interactive teaching, PHCC visits, office-based procedure exposure, and structured reflective writing). The bottom tier reports the evaluation, including favorable domain means (3.90 to 4.27 on a 5-point scale), high overall internal consistency (Cronbach’s alpha = 0.988), an 87.5% positive global rating, and Systems Thinking as the strongest independent predictor of perceived block value (beta = 0.727, *p* < 0.001).

COM353 sits within a spiral curriculum in which key themes are introduced early, revisited with greater depth, and consolidated during later clinical training. The six-year program is organized into pre-clerkship years (Years 1 to 3) and clinical clerkship years (Years 4 to 6), with formal hospital-based clerkships beginning in Year 4. During the pre-clerkship years, students complete integrated basic-science and organ-system blocks that build a working foundation in anatomy, physiology, pathology, and pharmacology, alongside limited and structured patient contact. Sustained, dedicated exposure to primary care and Family Medicine as a discipline first occurs in COM353 in the Fall semester of Year 3, which is the point at which the four pedagogical pillars are addressed explicitly and together. After COM353, students enter the hospital-based clerkships in Year 4, where the same pillars are intended to continue and deepen: experiential learning and reflection through ongoing clinical placements, work with clinical uncertainty and clinical reasoning as students take on greater diagnostic responsibility, and systems thinking as they move between primary, secondary, and tertiary settings and observe how the wider system connects. Specifically, the four pillars of COM353 are intended to be carried forward in two later opportunities: a Family Medicine clerkship rotation in Year 5, in which students return to primary care settings with greater clinical responsibility, and an elective one-month Family Medicine rotation taken by a subset of students during their Year 6 internship year. Figure 1 (top tier) situates the block within this trajectory, and the spiral is what allows a single Year 3 block to function as an integrative pivot rather than an isolated module.

That trajectory was not built in isolation. Two partnerships shape the block’s design and delivery, and they are described here as their canonical reference point in the manuscript. The curricular framework was developed by faculty in the Department of Family and Community Medicine at Alfaisal University, in academic collaboration with the Department of Family and Community Medicine, Temerty Faculty of Medicine, University of Toronto. This was an iterative consultative process conducted over several months rather than a single meeting: successive drafts of the block’s structure, reflective-writing components, and approach to clinical uncertainty were reviewed and refined through repeated academic exchange, while conceptual ownership and final decisions rested with the Alfaisal faculty team. Implementation of the experiential placement program was made possible through a clinical-systems collaboration with the Second Health Cluster in Riyadh, which provided coordinated student access to multiple Primary Health Care Centers across the city. This combined arrangement allowed integration of evidence-informed pedagogy with operational alignment to Saudi Arabia’s Vision 2030 PHC reform.

### 2.3 The educational intervention

In practical terms, COM353 is a four-week, three-credit-hour mandatory block delivered in the Fall semester of Year 3. It comprises six interlocking learning components: interactive didactic teaching, problem-based and case-based learning, experiential field visits, office-based procedures (OPS) and skills sessions, structured reflective practice, and self-directed learning, sequenced as iterative concept, experience, and reflection cycles across the 4 weeks. The didactic load consists of thirty timetabled lecture slots of approximately 50 minutes each, of which twenty-six are classroom-based interactive sessions and four are field-based experiential learning sessions (see below). In addition, students complete three problem-based learning cases, each delivered across two scheduled tutorial sessions, for a total of six PBL sessions; the three cases address Chronic Disease Management in the Community, Patient Safety and Health Promotion in Primary Care, and Digital Health and Innovation in Primary Care. The block concludes with a Block Review session and a final summative examination.

Pedagogically, the block stands on four ideas that the curriculum committee deliberately chose to foreground. The first is experiential and situated learning, which positions community PHCC visits as the primary site of insight rather than as illustrative add-ons. The second is reflection, which is treated as a developmental practice rather than an assessment artifact. The third is explicit engagement with clinical uncertainty, named openly in teaching rather than left implicit. The fourth is systems thinking, which in this context simply means the habit of seeing a single patient encounter as part of something larger, the clinic, the community, and the wider health system, rather than as an isolated event. In practical terms, a student practicing systems thinking asks not only what is wrong with this patient but also how this person reached care, what will happen after they leave, and how the surrounding services help or hinder their health. Implementation combines didactic teaching with PHCC visits, exposure to office-based procedures, and structured reflective writing.

#### 2.3.1 Didactic and interactive teaching

Core sessions introduce the discipline’s defining principles. Continuity of care, comprehensiveness, and coordination receive sustained attention, alongside first-contact access and preventive practice. Sessions also address scope of practice, patient-centered communication, and how primary care interfaces with specialty services through referral pathways. A recurring thread across the didactic strand is integration, weaving biomedical, psychosocial, and preventive considerations into the same encounter rather than treating them sequentially. Concretely, the classroom strand consists of approximately 26 interactive lecture sessions of 50 minutes each, organized around the four pedagogical pillars. Topics include the social determinants of health, value-based and team-based care, the structure of primary, secondary, and tertiary care, public-health regulation and policy, sustainability in healthcare, choosing wisely and deprescribing, vaccination across the lifespan, disease prevention and screening, communicable and non-communicable disease control, cancer screening, environmental health, patient safety, digital health and artificial intelligence in primary care, implicit bias, reproductive care, media advocacy, and dedicated sessions on developing clinical courage and Family Medicine as the discipline of generalism. Guest-expert sessions extend the range, and two integrating sessions on “clinical courage, building personal identity, and uncertainty” frame Family Medicine explicitly as the specialty of generalism. Teaching is interactive throughout. Faculty actively encourage questioning, model open discussion of uncertainty, and work through real-world scenarios with students rather than deliver content passively.

#### 2.3.2 Primary Health Care center visits

PHCC visits constitute the central experiential component of the block. In the Saudi health system, Primary Health Care Centers (PHCCs) are the Ministry of Health’s first-contact, community-based primary care clinics; the placements are therefore primary care visits, and the facility name PHCC denotes only the site. What makes them valuable for a Primary Health Care-oriented education is what students encounter there: not only first-contact clinical care, but preventive and public health functions, continuity and coordination, and the social context of the catchment population, the elements that connect a single primary care encounter to the wider system (6). Unlike short observational placements, they are designed as primary learning sites, exposing students to the realities of front-line care: comprehensive care across ages and conditions, preventive services and chronic disease management, continuity and follow-up, patient-centered encounters within time and system constraints, and clinical decision-making with incomplete data. Each student undertakes two supervised half-day field-learning sessions of approximately 3 hours each. The first is a Primary Health Care Center placement at a Second Health Cluster facility (the King Fahad Medical City Primary Health Care Center site), where students observe family-physician-led consultations, follow multi-concern encounters, and see preventive and chronic-disease care interwoven with acute presentations. The second is a community and pre-hospital field session at the Saudi Red Crescent Authority (SRCA), in which students observe prehospital and emergency-response systems and the way they coordinate with primary care. Each session is supervised on site by clinicians from the host service and accompanied by Department of Family and Community Medicine faculty. Visits are integrated through preparatory framing and guided reflection, so that experiences are interpreted analytically and connected to prior teaching rather than left as isolated observations. Site allocation, scheduling, and on-site supervision arrangements were coordinated through the Second Health Cluster, allowing students to rotate across multiple PHCCs in different catchment areas of Riyadh.

#### 2.3.3 Office-based procedures

The block also introduces students to common office-based procedures encountered in primary care, delivered as “how-to” skills sessions. This component is run as a structured curricular collaboration with the parallel Professional Skills (PRO 355) course, jointly coordinated by the Department of Family and Community Medicine and the Department of Clinical Skills, with sessions timed to align with COM353 delivery. The procedure curriculum covers needle and sharps injury safety, intramuscular, subcutaneous, and intradermal injections, basic cardiopulmonary resuscitation with automated external defibrillator use, shoulder joint injections, basic surgical skills (suturing and knot tying), and contraceptive methods. Students attend the sessions in the Clinical Skills Lab, where they observe demonstrations and then practice the procedures on simulation models under direct faculty supervision. The emphasis at this stage is on understanding how such procedures fit within first-contact, continuity-based care, and on safe technique, rather than on technical mastery, which is consolidated during later clerkships. Positioning this exposure in Year 3 is developmentally appropriate: it lets students connect a procedure to the whole patient and the wider service, in the same semester in which they are placed in Primary Health Care Centers, before they meet the same skills again, in greater depth, in the clinical years.

#### 2.3.4 Reflective writing

Reflection is built into the block as a pedagogical strategy, not an assessment artifact. After experiential activities, students complete structured writing exercises that prompt them to examine challenging encounters, experiences of uncertainty or discomfort, observations of patient-centered or fragmented care, emotional responses, and emerging professional values. Concretely, each student submits two separate structured reflections of 500 to 700 words, one after the PHCC visit and one after the SRCA visit, following a mandated four-part format. Section A is an objective description of the visit, covering the setting, services observed, roles of the healthcare professionals, and patient flow (approximately 150 words). Section B addresses key learning points, including what the student learned about the healthcare system, how care is coordinated, observed patient-safety practices, and any differences noticed between theory and practice (approximately 200 to 250 words). Section C is a personal reflection asking how the visit influenced the student’s understanding of family and community medicine, which professional behaviors were observed, which skills or attitudes the student now aims to develop, and how the experience connects to their future clinical training (approximately 150 to 200 words). Section D requires visual evidence (mandatory for the PHCC visit and optional for SRCA) with a strict no-identifiers rule. Reflection is developmental in intent but contributes to summative assessment: each reflection counts toward 5% of the block grade and is marked against a five-criterion rubric weighting completion and adherence to structure (20%), depth of reflection and insight (30%), link to course concepts (25%), professionalism and clarity (15%), and visual documentation where required (10%). Students receive individual rubric-based feedback, and the reflective component is explicitly designated as the route through which the block evaluates the Values and Professionalism competency domain, so that reflection functions as a developmental dialog rather than a one-way submission. The framing emphasizes psychological safety and honest engagement rather than performance.

#### 2.3.5 Clinical uncertainty and clinical courage

A distinctive feature of the block is the open normalization of uncertainty. Faculty discuss diagnostic and management uncertainty directly, model decision-making with incomplete information, and encourage students to articulate their own uncertainty without penalty. Clinical courage, the willingness to engage thoughtfully despite incomplete information, is named in teaching and reinforced through reflection. Importantly, uncertainty is not presented as a problem to be merely endured. It is paired explicitly with the teaching of clinical reasoning: faculty work through how to process an undifferentiated presentation, generating and refining a differential, deciding what can safely be observed over time, what must be excluded promptly, and when to seek help, so that tolerating uncertainty is coupled with concrete cognitive skills rather than left as an attitude alone. Two dedicated sessions on “developing clinical courage, building personal identity, and uncertainty: Family Medicine as the specialty of generalism” anchor this work in the timetable. Psychological safety is operationalized through small-group teaching with explicit ground rules, faculty modeling of “I do not know” in front of students, nonjudgmental post-visit debriefs after each experiential session, and an explicit assurance, communicated to students at the start of the block, that admitting uncertainty is welcomed and never penalized. The conceptual structure of the COM353 block, including its dual-partnership architecture, four pedagogical principles, core educational components, and key evaluation outcomes, is summarized in Figure 1.

### 2.4 Assessment and progression

COM353 is assessed through three components: a continuous-assessment Experiential Learning and Reflection portfolio worth 5% of the block grade, PBL-session performance worth 5%, and a summative final examination worth 90%. The final examination has two paper-based components designed to test different cognitive levels: a multiple-choice question paper worth 80% of the block grade, which assesses students’ understanding of the block modules and their critical thinking, and a short-answer question paper worth 10% of the block grade, which evaluates analytical skills and reasoning around clinical and population-level scenarios. To progress, students must achieve the College of Medicine standard pass mark of 65% in the final examination, in accordance with the university’s examination policy. The reflective portfolio is developmental in intent but contributes to the summative grade through the rubric described above, so that its formative purpose, structured feedback to deepen thinking, is preserved while it remains a graded component. The assessment design deliberately rewards thoughtful engagement with uncertainty and reflective reasoning rather than rote recall, keeping the assessment aligned with the block’s four pedagogical pillars.

### 2.5 Participants

The study population comprised all third-year medical students enrolled in COM353 during January 2026 (*N* = 126). At block conclusion, eligible students received an anonymous, voluntary online survey via official course channels. Participation was explicitly described as voluntary, anonymous, and unrelated to academic assessment or standing. No incentives were offered. With 96 responses and six predictors in the planned regression analysis, the sample provided approximately 80% power to detect medium effects (f-squared = 0.15) at alpha = 0.05 (17).

### 2.6 Survey instrument

We developed the instrument by adapting items from validated tools and supplementing them with block-specific content. The adaptation was grounded in two activities: a focused review of the literature on educational-environment and reflection instruments, which identified the DREEM and GRAS as the best-validated source tools mapping onto our constructs, and an internal expert-consultation process within the Department’s curriculum committee. Two source tools were drawn on. The DREEM, validated for assessing perceptions of the educational environment (15), supplied items that mapped onto our learning environment domain. The GRAS, validated for reflective capacity (16), informed the reflection domain. Source-tool items were retained where they aligned with our block constructs and were contextually adapted where wording referred to specialty-agnostic clinical experiences rather than Family Medicine. New items were written for areas the source tools did not cover well, especially clinical uncertainty, clinical courage, and systems thinking. Item generation moved through three stages. The Department’s curriculum committee began by defining the constructs against the four pedagogical principles of the block. Items were then drafted, drawing on the validated tools and on Hillen et al.’s tripartite uncertainty framework (11) and the Health Systems Science framework described by Gonzalo et al. (18). Four faculty content experts then reviewed each item independently, rating it for clarity, relevance, and construct alignment, and disagreements were resolved by consensus. Items unanimously judged ambiguous or redundant at this stage were either rephrased or removed.

The final questionnaire ran to 42 Likert-scale items, organized across seven domains. Items 1 through 7 addressed Learning Environment and Teaching Quality, while Items 8 through 14 covered Family Medicine and PHC Competencies. Items 15 to 20 dealt with Experiential Learning and PHC Visits, and Items 21 to 26 with Clinical Courage and Uncertainty Tolerance. Reflection and Professional Identity Formation occupied Items 27 to 32, Systems Thinking and Community Orientation Items 33 to 37, and Overall Impact and Readiness Items 38 to 42. The survey also included a single global rating of the block on a Poor-to-Excellent scale and one optional open-ended question. Likert items used a five-point response format (Strongly Disagree, Disagree, Neutral, Agree, Strongly Agree), coded 1 through 5. The complete instrument, with all 42 items grouped by domain together with the global rating and the open-ended prompt, is provided as Supplementary File 2.

Because Primary Health Care competence is central to this Research Topic, the content of the Family Medicine and PHC Competencies domain (Items 8 to 14) is summarized here rather than confined to the supplement. These items asked students to rate their understanding of the core principles of Family Medicine, the role of Primary Health Care within the health system, continuity of care, comprehensive care across age groups and conditions, preventive care and health promotion, the family physician’s role as a first-contact clinician, and the scope of primary care practice.

### 2.7 Data collection

The survey was administered online using a secure, institutionally approved platform immediately after block completion. Responses were anonymous to the research team. The platform captured institutional email addresses solely for one-time access control, to prevent duplicate submissions; these identifiers were automatically stripped from the dataset before any analysis, so that no response could be linked to an individual student. No names, student numbers, or other direct identifiers were collected, and the investigators received only de-identified data.

### 2.8 Ethical considerations

The study was reviewed and approved by the Institutional Review Board of Alfaisal University, College of Medicine (approval number IRB20362-2, approved November 2025). Third-year medical students are a potentially vulnerable group in educational research, because a perceived link between participation and academic standing could create pressure to take part. The study was designed to remove that pressure. Because the study posed minimal risk and used an anonymous, voluntary educational survey unconnected to summative assessment, the Board approved the use of implied consent. Students received a study information sheet describing the purpose, voluntary nature, anonymity safeguards, and right to decline without consequence. The information sheet stated explicitly that the decision to participate or not would have no bearing on their grades, their assessment, or their relationships with teaching staff or the university, and that no member of the teaching faculty would be able to identify who had responded; consent was inferred from completion of the survey. No incentives were offered, and no data were used for academic evaluation. No potentially identifiable images or individual-level data are presented in this article, and participant confidentiality was maintained throughout.

### 2.9 Data analysis

Likert responses were coded numerically (1 to 5), with higher scores indicating more favorable perceptions. Data were screened for completeness and for response patterns suggesting inattentive responding; none were detected. All analyses were performed in IBM SPSS Statistics version 29 (IBM Corp., Armonk, NY). Descriptive statistics were calculated at item and domain levels. Domain composite scores were computed as means of the constituent items. Internal consistency was assessed using Cronbach’s alpha, which summarizes how consistently the items within a domain move together: an alpha near 1.0 means students who scored one item highly tended to score the other items in that domain highly as well, indicating the items are measuring the same underlying idea, with values at or above 0.70 considered acceptable, 0.80 good, and 0.90 excellent (19); the average inter-item correlation was also computed to flag potential item redundancy. One-sample *t*-tests compared each domain mean against the scale midpoint (3.0), and Cohen’s d was computed for effect size (17). We report *p* < 0.001 for all such comparisons because every domain mean differed from the midpoint at that level.

Inter-domain Pearson correlations were calculated to examine the structural coherence of the survey. To identify which domains independently predicted overall block evaluation, we fitted a multiple linear regression using six domain composites (Learning Environment, Family Medicine Competencies, PHC Visits, Uncertainty Tolerance, Reflection, and Systems Thinking) as predictors and Item 42 (“Overall, COM353 was a valuable and effective block”) as the outcome. Model assumptions were checked through residual plots and the Durbin-Watson statistic, and multicollinearity was assessed using variance inflation factors (VIF), with VIF < 10 considered acceptable and VIF < 5 ideal. Standardized betas are reported alongside unstandardized coefficients to support interpretation. Significance was set at alpha = 0.05 (two-tailed).

## 3 Results

### 3.1 Response rate and sample characteristics

Of 126 third-year students enrolled in the January 2026 cohort, 96 returned completed surveys, yielding a response rate of 76.2%. All 96 responses were complete on all Likert items; no imputation was required. The achieved rate exceeds both the pooled health sciences education benchmark of 67% reported by Wilson et al. across 360 studies (20) and the 70% threshold commonly applied to support generalizable findings in survey research.

### 3.2 Domain-level findings

Students reported favorable perceptions across the board. Mean scores by domain ran from 3.90 to 4.27 on the 5-point scale, and every mean exceeded the scale midpoint of 3.0 in one-sample *t*-tests (all *p* < 0.001). Effect sizes relative to the midpoint were large by conventional standards, ranging from Cohen’s d = 0.92 to 1.58 (17). Agreement rates (Agree or Strongly Agree combined) varied between 63.7 and 80.7%. The instrument as a whole showed excellent internal consistency (Cronbach’s alpha = 0.988), and each domain alpha sat in the excellent range (0.940 to 0.974). The average inter-item correlation across the full instrument was 0.65, somewhat above the 0.15 to 0.50 range usually recommended for unidimensional scales (19). That value tells us the seven domains share substantial common variance, and that some items overlap conceptually within an integrated curricular evaluation, a point we return to in the Discussion.

Domain-level statistics appear in Table 1. Family Medicine and PHC Competencies stood at the top, with the highest mean (4.27, SD 0.81) and the highest agreement rate (80.7%). At the item level within that domain, perceived growth in continuity of care drew 84.4% agreement, and the item on the family physician’s role as first-contact clinician drew 81.2%. Systems Thinking followed closely, with the second-highest mean (4.20, SD 0.86) and 76.9% agreement. Experiential Learning produced a domain mean of 4.10 (SD 0.96, 71.9% agreement), and Clinical Courage and Uncertainty Tolerance produced 4.01 (SD 0.84, 67.5% agreement). The single item asking about thoughtful clinical reasoning rather than defensive medicine drew 76.0% agreement. Reflection and Professional Identity recorded the lowest domain mean (3.90, SD 0.98) and the lowest agreement rate (63.7%), with the largest share of neutral responses, although the domain still trended favorable overall.

**Table 1:** Domain-level descriptive statistics, internal consistency, and one-sample *t*-tests against the scale midpoint (*N* = 96).

Domain	Items	*M* (SD)	Agree %	Cohen’s d	Alpha	*p*
Learning Environment	1–7	4.03 (0.88)	71.7	1.17	0.940	<0.001
Family Medicine and PHC Competencies	8–14	4.27 (0.81)	80.7	1.58	0.974	<0.001
Experiential Learning and PHC Visits	15–20	4.10 (0.96)	71.9	1.15	0.964	<0.001
Clinical Courage and Uncertainty Tolerance	21–26	4.01 (0.84)	67.5	1.20	0.953	<0.001
Reflection and Professional Identity	27–32	3.90 (0.98)	63.7	0.92	0.971	<0.001
Systems Thinking and Community Orientation	33–37	4.20 (0.86)	76.9	1.39	0.965	<0.001
Overall Impact and Readiness	38–42	4.06 (0.88)	71.7	1.20	0.958	<0.001

The table has been simplified from the original submission: the one-sample *t*-statistic column has been removed for readability, retaining the mean, standard deviation, agreement rate, effect size, internal-consistency reliability, and *p*-value for each domain. M, mean; SD, standard deviation; Agree %, combined Agree and Strongly Agree responses across all items in the domain; Cohen’s d computed against the scale midpoint of 3.0; Alpha, Cronbach’s alpha; *p*, significance of the one-sample *t*-test against the midpoint of 3.0. All one-sample *t*-tests were significant at *p* < 0.001 (*t*-statistics ranged from 8.99 to 15.44, df = 95).

**Figure 2:**
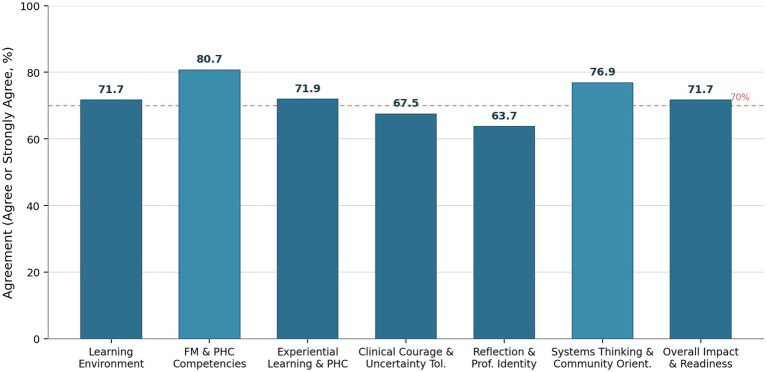
Student agreement by educational domain. Bars show the percentage of students selecting Agree or Strongly Agree (combined) for each of the seven domains; the dashed line marks the 70% reference level. Agreement was highest for Family Medicine and PHC Competencies (80.7%) and lowest for Reflection and Professional Identity (63.7%).

The distribution of agreement across the seven domains is shown visually in Figure 2, which presents the combined Agree and Strongly Agree percentage for each domain against a 70% reference line.

### 3.3 Overall block rating

When asked for a single global rating, 36.5% of students rated the block Excellent, 22.9% Very Good, and 28.1% Good, yielding a combined positive rating of 87.5%. Only 9.4% rated it Fair and 3.1% Poor. The mean global rating was 3.80 (SD 1.13) on the 1 (Poor) to 5 (Excellent) scale.

### 3.4 Inter-domain correlations

Pearson correlations among the seven domains were uniformly positive and substantial, ranging from r = 0.544 to 0.918 (all *p* < 0.001; Table 2). Table 2 matters because it shows how tightly the domains hang together: rather than measuring seven unrelated things, the instrument captures a set of closely linked perceptions, which is what one would expect if students experienced the block as a single integrated whole. The strongest association linked Systems Thinking with Overall Impact and Readiness (*r* = 0.918). Reflection produced the most modest correlations (*r* = 0.544 to 0.823), though every coefficient remained large. The pattern is consistent with structural coherence across the block: students who reported favorable perceptions in one domain tended to do the same elsewhere, suggesting the integrated curricular design was experienced as a connected whole rather than as discrete modules.

**Table 2:** Inter-domain Pearson correlation matrix (*N* = 96).

Domain	(1)	(2)	(3)	(4)	(5)	(6)	(7)
(1) Learning Environment	1.000						
(2) FM and PHC Competencies	0.831	1.000					
(3) PHC Visits	0.644	0.680	1.000				
(4) Uncertainty Tolerance	0.867	0.803	0.677	1.000			
(5) Reflection	0.756	0.622	0.544	0.823	1.000		
(6) Systems Thinking	0.848	0.874	0.741	0.875	0.697	1.000	
(7) Overall Impact	0.853	0.821	0.718	0.867	0.779	0.918	1.000

All correlations significant at *p* **<** 0.001. PHC, Primary Health Care; FM, Family Medicine.

### 3.5 Predictors of overall block evaluation

Multiple linear regression with six domain composites predicting overall evaluation (Item 42) accounted for 78.2% of variance (R-squared = 0.782, adjusted R-squared = 0.767; *F*(6, 89) = 53.25, *p* < 0.001). Residual diagnostics gave no cause for concern: the Durbin-Watson statistic was 1.77, suggesting no problematic autocorrelation, and variance inflation factors fell below 10 for every predictor (range 2.26 to 7.64). Uncertainty Tolerance and Systems Thinking sat at the high end of that range, with VIFs of 7.64 and 7.42, respectively. These figures indicate that some predictors share substantial variance, consistent with the integrated curricular design, but the values do not breach the threshold at which collinearity would invalidate the regression. Three domains reached independent significance. Systems Thinking carried much the strongest weight (beta = 0.727, *p* < 0.001), with Reflection (beta = 0.295, *p* = 0.002) and Learning Environment (beta = 0.293, *p* = 0.013) following. The remaining three predictors, Family Medicine Competencies, PHC Visits, and Uncertainty Tolerance, did not reach independent significance after adjustment, although each had a positive bivariate association with the outcome. The Family Medicine Competencies result is the most striking. Despite producing the highest absolute scores in the study, the domain did not predict overall evaluation independently when the other domains were held constant. The most plausible reading is shared variance with Systems Thinking, with which it correlated at r = 0.874. Full regression coefficients appear in Table 3.

**Table 3:** Multiple linear regression: domain composites predicting overall block evaluation (Item 42; *N* = 96).

Predictor	B	SE	Beta	*t*	*p*	95% CI	VIF
(Constant)	−0.118	0.275	n/a	−0.43	0.668	−0.66 to 0.43	n/a
Learning Environment	0.334	0.132	0.293	2.53	0.013	0.07 to 0.60	5.47
Family Medicine Competencies	−0.245	0.137	−0.197	−1.79	0.077	−0.52 to 0.03	4.96
PHC Visits	0.064	0.078	0.061	0.82	0.416	−0.09 to 0.22	2.26
Uncertainty Tolerance	−0.283	0.164	−0.236	−1.73	0.087	−0.61 to 0.04	7.64
Reflection	0.303	0.093	0.295	3.27	0.002	0.12 to 0.49	3.33
Systems Thinking	0.847	0.157	0.727	5.39	<0.001	0.54 to 1.16	7.42

Outcome: Item 42 (“Overall, COM353 was a valuable and effective block”). Model: R-squared = 0.782, adjusted R-squared = 0.767; *F*(6, 89) = 53.25, *p* **<** 0.001. Durbin-Watson = 1.77. B, unstandardized regression coefficient; SE, standard error; Beta, standardized regression coefficient; *t*, *t*-statistic; CI, confidence interval; VIF, variance inflation factor. All VIFs are below 10, indicating multicollinearity does not invalidate the regression.

## 4 Discussion

### 4.1 Principal findings

Across all seven educational domains, students reported favorable perceptions, with effect sizes from 0.92 to 1.58 relative to the scale midpoint, every one of them large by conventional standards. The 87.5% positive global rating and the very high instrument reliability (alpha = 0.988) together suggest that the block’s integrated design resonated with third-year students at this critical point in their training. The most striking result, however, is not an absolute score. It is the pattern in the regression: Systems Thinking emerged as the strongest predictor of overall block evaluation, followed by Reflection and Learning Environment. Students who left the block with an integrated view of healthcare as a system, and who engaged meaningfully with reflective practice, perceived the greatest educational value, even when their absolute scores in other domains were not the highest.

### 4.2 Learning environment and educational climate

The favorable learning environment perceptions sit comfortably within regional benchmarks. Awawdeh et al., applying the DREEM at King Saud bin Abdulaziz University for Health Sciences in Riyadh, reported a total score of 125.88 out of 200, with third-year students producing the highest perception scores (mean 132.23) (15). In our data, 83.3% of students agreed on items addressing psychological safety and faculty encouragement of discussion, a result consistent with that favorable range. The block’s emphasis on psychological safety appears justified by accumulating evidence. McClintock et al., synthesizing 52 articles on psychological safety in medical education, found it consistently defined as the belief that the learning environment permits intellectual risk-taking (21). Torralba et al., in a Veterans Affairs study of more than 13,000 residents, demonstrated that psychological safety predicted overall satisfaction with the learning environment (22). The independent predictive role of Learning Environment in our regression (beta = 0.293) adds further support, although the direction of the relationship cannot be settled definitively in cross-sectional data.

### 4.3 Family medicine and primary care competencies

The Family Medicine Competencies domain achieved the highest mean and agreement rate, indicating that the block’s core content was successfully transmitted to most students. Items on continuity of care (84.4%) and on the family physician as first-contact clinician (81.2%) performed especially well, both speaking directly to discipline-defining concepts. These two items map onto two of Barbara Starfield’s four classic principles of Primary Care, first-contact access and longitudinality, which the World Health Organization treats as central to PHC-based service delivery (4, 6).

Comparisons with regional and international benchmarks are encouraging, though they must be read with the level of training in mind. Our study evaluated undergraduate medical students during a pre-clerkship block, whereas much of the comparable Family Medicine literature describes postgraduate trainees or residents; the two groups differ in clinical experience, and direct numeric comparison is therefore indicative rather than exact. Almutairi et al., surveying 183 graduates of Saudi Board Family Medicine programs, reported a perceived achievement of SaudiMED-FM 2020 competencies at 23.06 of 30 (roughly 77%), with no significant difference between three- and four-year programs (23), a postgraduate residency benchmark rather than an undergraduate one. Turkeshi et al., in their systematic review of 64 Family Medicine clerkship studies, documented consistently high satisfaction with family medicine teaching across diverse settings, predominantly at the undergraduate clerkship level (7). Our findings sit within this range while addressing a distinct stage of training, namely an undergraduate block earlier than the clerkship rather than residency. The Saudi context further amplifies the relevance: Vision 2030 places “a family physician for every family” at the center of the country’s strategy for universal health coverage (24), and undergraduate interventions that strengthen FM understanding contribute directly to that broader health system goal.

### 4.4 Experiential learning and PHC placements

Students valued the PHCC visits as meaningful opportunities to apply theory in authentic settings (mean 4.10; 71.9% agreement; d = 1.15). The data support positioning community-based placements as central learning experiences rather than supplementary observations. Community-based and longitudinal placements in general practice are now a well-established component of undergraduate curricula across Europe and beyond, and the discipline’s professional bodies regard substantial, sustained exposure to general practice as an ideal rather than a token attachment (2, 3). Achieving that ideal, however, is not straightforward. Sufficient placement capacity depends on securing enough clinical sites and trained community mentors, and this remains a recurring practical constraint on Primary Care education internationally (25). The infrastructure that made our placements possible, a clinical-systems partnership with the Second Health Cluster in Riyadh, was therefore not incidental but central: high-quality experiential placement requires negotiated access to real service-delivery sites and supervising clinicians, and where that access cannot be brokered, community-based teaching is difficult to sustain. Longer or longitudinal community placements have been associated with positive effects on graduates’ orientation toward generalist careers (25, 26), which further strengthens the case for protecting placement capacity. The findings align with established educational theory. Wijnen-Meijer et al. described a successful application of Kolb’s experiential learning cycle in family medicine, with 92% of students reporting that learning goals were achieved and showing particular enthusiasm for discussion of personally encountered cases (12). Orsmond et al., applying Lave and Wenger’s situated learning framework, found that early clinical practice in primary care supports professional identity formation through reflection-in-action (27). Dornan et al., in their experience-based learning model, identified “supported participation” as the core condition for effective workplace learning (28). The COM353 design, which frames PHCC visits as primary learning sites requiring analytical interpretation rather than passive observation, embodies these principles directly.

### 4.5 Clinical uncertainty tolerance and clinical courage

About two-thirds of students reported greater comfort with clinical uncertainty after the block (mean 4.01; 67.5% agreement; d = 1.20). The item on thoughtful clinical reasoning rather than defensive medicine drew 76.0% agreement, suggesting that students grasped the distinction between productive uncertainty tolerance and inappropriate hesitancy. Hillen et al. described uncertainty as arising from several distinct sources (11). One is ambiguity, where information is inadequate. Another is probability, where outcomes are indeterminate. A third is complexity, where the relevant factors are tightly interconnected. Primary Care offers natural ground on which to develop competency in working through all three, given its undifferentiated presentations and longitudinal relationships. Evidence that uncertainty tolerance is modifiable is now substantial. In a scoping review, Patel et al. reported that 22 of 24 studies documented positive impact, with effective interventions including problem-based learning, medical humanities, simulation, and reflection (10). Taylor et al. demonstrated sustained improvement among Family Medicine residents who completed a curriculum that paired readings with reflective writing and group discussion (29). The COM353 approach sits within this evidence base. Uncertainty is named explicitly in teaching, faculty model decision-making with incomplete information, and reflection serves as the integrative thread. The clinical courage construct draws theoretical support from Reis-Dennis et al., who argued that three “corrective virtues” can help physicians work through uncertainty: courage (countering the impulse to flee), diligence (mitigating premature closure), and curiosity (driving productive engagement with the unknown) (30). Our high tolerance scores may, then, reflect cultivated virtues rather than passive acceptance.

### 4.6 Reflection and professional identity formation

Reflection produced the lowest domain mean (3.90) and the lowest agreement rate (63.7%), yet emerged as the second strongest independent predictor of overall block evaluation (beta = 0.295). This pattern, low absolute scores combined with high predictive weight, is one of the more counterintuitive findings of the study and warrants careful interpretation. Two readings are plausible. The first is dispositional: students who arrive at the block already inclined toward reflective engagement may benefit disproportionately from the curricular components that match their orientation, even though group means stay modest because not every student develops reflective habits during a brief exposure. The second reading is pedagogical: meaningful reflection may be harder to achieve than other learning gains, but when it does take hold, its integrative effect across domains is substantial. Both readings argue for protected curricular space and faculty development around reflective pedagogy, rather than for treating reflection as an add-on. The finding aligns with comparable patterns in the broader medical education literature. GRAS-based studies show variable means: Andersen et al. reported a Danish student mean of 76.5% (16), and Hafiz et al. described a correlation of r = 0.403 between reflective thinking and clinical decision-making in medical faculty students (31), close to our regression coefficient. Quality of reflection appears to matter more than quantity. Winkel et al., in a systematic review of reflection as a learning tool in graduate medical education, found that reflective capacity develops vertically, with superficial engagement potentially deepening into critical synthesis (32). Students who engage meaningfully, even briefly, may derive disproportionate benefit. The professional identity literature deepens this picture. Cruess et al. describe professional identity as a self-representation built over time through internalization of professional characteristics, values, and norms (33). Sternszus et al. recently emphasized that socialization involves active, critical engagement with professional norms, often facilitated through group reflective exercises in which learners share stories and explore interpretations (34). Students who engage with COM353’s reflective components may experience accelerated identity formation, which in turn contributes to overall satisfaction with the block.

### 4.7 Systems thinking as the integrative competency

That Systems Thinking emerged as the dominant predictor (beta = 0.727) is, for our team, the most consequential finding. The domain achieved the second-highest mean (4.20) and 76.9% agreement, with strong responses on understanding healthcare as a system (79.2%), community-oriented care (78.1%), and interprofessional collaboration (77.1%). This pattern is also reflected visually in Figure 1, where systems thinking is positioned as both a pedagogical principle and the strongest quantitative predictor of perceived overall block value. Systems thinking is well suited to Primary Care precisely because Primary Care sits at the meeting point of clinical medicine and the wider determinants of health. A family physician routinely manages not just disease but the complex system surrounding a patient: the social determinants of health that shape who becomes ill and who recovers, and the question of access to care that determines who reaches services in the first place (4, 35). Teaching students to see this larger system, rather than the isolated consultation, is part of what the renewed global Primary Health Care agenda asks of contemporary curricula (5, 6). One block-level item in this domain specifically addressed equity, access, and the social determinants of health, and it drew favorable agreement, suggesting students did connect individual encounters to these system-level forces. The result is consistent with Health Systems Science (HSS) literature that positions systems thinking as the linking domain that ties other competencies together. Gonzalo et al. established the three-pillar framework, basic science, clinical science, and health systems science, with systems thinking uniquely connecting HSS topics (patient safety, quality improvement, value-based care) to cross-cutting competencies such as leadership, collaboration, and evidence-based practice (18). In a later piece, Gonzalo et al. proposed “systems citizenship” as the sixth wave of medical professionalism: physicians who view themselves within the larger health system context and accept a duty to continuous healthcare improvement (36). Students who develop this integrated perspective likely perceive greater coherence across their educational experiences, which would explain the strong predictive relationship in our data. Khanna et al. identified systems thinking as the cornerstone of systems-based practice, the foundation of curricular coherence (37). The same mechanism appears to operate here: students who grasp how educational components connect may perceive each individual block as more valuable than students who experience the curriculum as a series of disconnected modules. This interpretation has practical consequences: a curriculum that wants to maximize perceived value may do so most efficiently by teaching students to see the system, with other gains following from that vantage point. This is also where a primary care curriculum connects upward to Primary Health Care as a system: clinicians trained to look beyond the single consultation to prevention, coordination, and the determinants of health are the workforce a Primary Health Care-oriented system depends on, and building that capacity is the contribution undergraduate primary care education makes to the wider goal (5, 6).

### 4.8 Methodological considerations

Four points deserve careful interpretation. First, the 76.2% response rate exceeds widely used thresholds for survey-based educational research. Wilson et al.’s meta-analysis of 360 health sciences education surveys produced a pooled response rate of 67% (20), and Ericson et al. identified 70% as the threshold for generalizable findings in graduate medical education research (38). Our rate falls comfortably above both. Second, the very high overall alpha (0.988) and average inter-item correlation (0.65) warrant transparent interpretation. Tavakol and Dennick noted that values exceeding 0.90 may signal item redundancy (19), and inter-item correlations above 0.50 often indicate that scales measure overlapping rather than fully distinct constructs. The pattern in our data likely reflects a combination of two factors: genuine conceptual coherence across an integrated block in which students who appreciate one element also appreciate related ones, and instrument design in which related constructs (for example, Family Medicine Competencies and Systems Thinking) draw on overlapping cognitive content. Future iterations should test reduced item sets through exploratory factor analysis or item-response modeling, with the aim of preserving construct breadth while trimming redundancy. Third, ceiling effects were observed across domains. Between 25 and 42% of respondents reached near-ceiling domain scores (mean at or above 4.85), with the largest concentrations in Family Medicine Competencies (41.7%) and Systems Thinking (35.4%). Restricted upper-end variance can attenuate true effect-size estimates and bias predictive relationships toward the variables with greater spread, a possibility consistent with the relatively higher predictive role of Reflection (which had the lowest mean and the widest distribution) compared with Family Medicine Competencies (which had the highest mean and substantial ceiling). Fourth, the effect sizes are large by Cohen’s standards (17), but Kraft has cautioned that Cohen’s benchmarks are derived from psychological research and may not transfer cleanly to education research, where 0.20, 0.40, and 0.60 may better represent small, medium, and large effects (39). Even by the more demanding education-specific standard, our effects remain exceptionally large.

### 4.9 Limitations

Several limitations apply. The cross-sectional design captures perceptions at a single time point and cannot assess durability or translation to clinical performance. Self-report data carry the risk of social desirability bias despite anonymity. Because the survey was voluntary and administered online, the findings are also subject to participation and non-response bias: the 23.8% of eligible students who did not respond may have held systematically different views, and online voluntary surveys are known to over-represent more engaged or more favorably disposed respondents, which may inflate the favorable perceptions reported here and limits the reproducibility of the exact estimates. The absence of a comparison group limits causal inference; we cannot say whether students would have reported similar gains under alternative curricular arrangements, and this also constrains the internal validity of any inference that the block itself produced the perceptions observed. The instrument represents adaptation rather than *de novo* validation, although adapted items were derived from validated tools and the achieved reliability supports their internal consistency. Ceiling effects across most domains restrict variance available for regression and may attenuate effect-size estimates. The single-institution design at a private Saudi medical school limits external validity and generalizability to other contexts, particularly public institutions, other regions, or different cultural and health-system settings. Two further limitations concern scope of measurement. The evaluation was purely quantitative and did not include a qualitative component, such as student interviews or focus groups or analysis of the reflective writing, that could have explained why systems thinking carried such predictive weight or captured experiences the closed items missed. In addition, the study measured only student perceptions and did not gather the perspectives of the teaching faculty, clinical preceptors, or PHCC staff, whose views would give a more complete account of how the block functioned. Finally, we measured perceptions rather than objective competency. Perceptions are valuable for educational evaluation, but they are not a substitute for direct competence assessment, which we did not undertake here.

### 4.10 Implications and future directions

For educators, three implications follow. First, integrating community-based placements with structured reflection and explicit attention to clinical uncertainty appears to produce favorable outcomes across multiple educational domains. Second, systems thinking deserves particular curricular emphasis, not only as a competency in its own right but as the integrative thread that helps students experience the curriculum as coherent. Third, the predictive role of reflection (beta = 0.295) despite its lower absolute scores highlights the value of investing in reflective pedagogy quality rather than quantity. A further implication concerns measurement itself. Robust, validated instruments for evaluating Primary Care and Family Medicine education are scarce: the field leans heavily on a small number of established tools, principally the DREEM for educational climate and the GRAS for reflective capacity (15, 16, 40), and no widely validated instrument captures the full breadth of an integrated block spanning systems thinking, clinical uncertainty, and community orientation. This scarcity is itself a limitation for the field and points to a clear need for the deliberate development and psychometric validation of new instruments designed for contemporary integrated Primary Care curricula. Future research should pursue longitudinal follow-up to test whether the perceived gains observed here translate into clerkship performance, residency choice, and ultimate practice patterns. Multi-institutional studies would help test generalizability across settings, and instrument refinement through factor analysis could trim redundancy revealed by the high overall reliability. Mixed-methods designs that pair survey data with reflective writing analysis, and that incorporate the perspectives of teaching staff and clinical preceptors, would also help characterize the quality of reflection and the functioning of the block more fully than student self-report alone allows.

### 4.11 Academic collaboration as design infrastructure

The block evaluated here was not designed in isolation. Two distinct partnerships shaped its existence. The first was an academic collaboration between the Alfaisal University Department of Family and Community Medicine and the Department of Family and Community Medicine, Temerty Faculty of Medicine, University of Toronto, in which curricular structure, reflective pedagogy, and engagement with clinical uncertainty were informed by long-standing Canadian experience in undergraduate Family Medicine teaching while remaining locally led. This was an iterative consultative partnership rather than a single meeting or a turnkey curriculum transfer; conceptual ownership rested with Alfaisal faculty, adaptation was governed by Saudi PHC realities, learner culture, and language, and the design integrated regional priorities such as Vision 2030 workforce goals. The second was a clinical-systems partnership with the Second Health Cluster in Riyadh, which made the experiential placement program possible by coordinating student access across multiple PHCCs in different catchment areas of the city. The Cluster’s involvement aligned the placement program with operational PHC reform and gave students authentic exposure to the kind of front-line settings in which Saudi Arabia’s expanding family medicine workforce will practice.

Together, these arrangements illustrate how academic collaboration can operate at two levels simultaneously: institution-to-institution at the design level and academic-to-health-system at the implementation level. From a Sustainable Development Goal perspective, the COM353 case shows SDG 17 (partnerships for the goals) operating as the enabling infrastructure for SDG 3 (good health and well-being), with university-led pedagogy meeting health-system-led service delivery in the same student experience. The favorable educational outcomes reported here suggest that this dual-partnership model is a viable template for undergraduate FM education in settings where PHC strengthening and workforce expansion are concurrent national priorities. Replicating the model elsewhere would not require equivalent partner institutions; what it requires is the structural distinction between design partnership and delivery partnership, and explicit investment in both.

## 5 Conclusion

A purposefully integrated Family Medicine and Primary Care block that emphasizes experiential learning, reflective practice, and explicit work with clinical uncertainty can produce favorable educational outcomes across multiple domains in third-year medical students. The finding that systems thinking most strongly predicts overall block value, ahead of reflection and learning environment quality, offers practical guidance for curricular design: helping students grasp healthcare as a connected system may amplify the perceived value of every other component. Large effect sizes across domains suggest that this kind of integrated approach, in which community-based placements, structured reflection, and engagement with uncertainty are knit together rather than scattered, can meaningfully advance undergraduate students’ readiness for clinical practice.

## Data Availability

The de-identified data supporting the conclusions of this article are available from the corresponding author on reasonable request.

## Supplementary material

Supplementary File 1 STROBE checklist for cross-sectional studies, completed for this study.

Supplementary File 2 COM353 post-block student feedback questionnaire. The full 42-item instrument grouped by the seven domains, together with the single global rating item and the open-ended prompt.

## References

[1] Parekh R Jones MM Singh S Yuan JSJ Chan SCC Mediratta S . Medical students' experience of the hidden curriculum around primary care careers: a qualitative exploration of reflective diaries. BMJ Open. (2021) 11:e049825. doi: 10.1136/bmjopen-2021-049825, 34326054 PMC8323369

[2] Windak A Rochfort A Jacquet J. The revised European Definition of General Practice/Family Medicine. A pivotal role of One Health, Planetary Health and Sustainable Development Goals. Eur J Gen Pract. (2024) 30:2306936. doi: 10.1080/13814788.2024.2306936 38334099 PMC10860453

[3] WONCA Europe. The European Definition of General Practice/Family Medicine: 2023 Edition. Brussels: WONCA Europe (2023).

[4] Starfield B Shi L Macinko J. Contribution of primary care to health systems and health. Milbank Q. (2005) 83:457–502. doi: 10.1111/j.1468-0009.2005.00409.x, 16202000 PMC2690145

[5] World Health Organization, United Nations Children's Fund (UNICEF). Declaration of Astana. Global Conference on Primary Health Care, Astana, Kazakhstan. Geneva: World Health Organization (2018).

[6] World Health Organization. In: United Nations Children's Fund (UNICEF), editor. Operational Framework for Primary Health Care: Transforming Vision into Action. Geneva: World Health Organization (2020)

[7] Turkeshi E Michels NR Hendrickx K. Impact of family medicine clerkships in undergraduate medical education: a systematic review. BMJ Open. (2015) 5:e008265. doi: 10.1136/bmjopen-2015-008265 PMC4538263 26243553

[8] Camarata T Spizzieri A Kulkarni A. Undergraduate medical education amidst volatility, uncertainty, complexity, ambiguity. Front Med. (2025) 12:1666718. doi: 10.3389/fmed.2025.1666718, 41229514 PMC12602499

[9] Kerr AM Thompson CM. Medical students’ reactions to uncertainty during clinical rotations Fam Med. (2022) 54:285–289. doi: 10.22454/FamMed.2022.947719 35421243

[10] Patel P Hancock J Rogers M Pollard SR. Improving uncertainty tolerance in medical students: a scoping review. Med Educ. (2022) 56:1163–73. doi: 10.1111/medu.14873, 35797009 PMC9796811

[11] Hillen MA Gutheil CM Strout TD Smets EMA Han PKJ. Tolerance of uncertainty: conceptual analysis, integrative model, and implications for healthcare. Soc Sci Med. (2017) 180:62–75. doi: 10.1016/j.socscimed.2017.03.024, 28324792

[12] Wijnen-Meijer M Brandhuber T Schneider A Berberat PO. Implementing Kolb's experiential learning cycle by linking real experience, case-based discussion and simulation. J Med Educ Curric Dev. (2022) 9:23821205221091511. doi: 10.1177/23821205221091511 35592131 PMC9112303

[13] Heydari S Beigzadeh A. Medical students’ perspectives of reflection for their professional development. BMC Med Educ. (2024) 24:1399. doi: 10.1186/s12909-024-06401-2 39614257 PMC11607811

[14] Shikino K Yamauchi K Araki N Ozaki N Kamata Y Aoki S . Impact of community-oriented medical education on medical students' perceptions of community health care: qualitative study. JMIR Med Educ. (2026) 12:e84406. doi: 10.2196/84406, 41554117 PMC12865343

[15] Awawdeh M Alosail LA Alqahtani M Almotairi A Almikhem RN Alahmadi RA . Students' perception of the educational environment at King Saud bin Abdulaziz University for Health Sciences using DREEM tool. BMC Med Educ. (2024) 24:42. doi: 10.1186/s12909-023-05004-7, 38191423 PMC10775488

[16] Andersen NB O'Neill L Gormsen LK Hvidberg L Morcke AM. A validation study of the psychometric properties of the Groningen reflection ability scale. BMC Med Educ. (2014) 14:214. doi: 10.1186/1472-6920-14-214 25304774 PMC4286925

[17] Cohen J. Statistical Power Analysis for the Behavioral Sciences. 2nd ed. Hillsdale, NJ: Lawrence Erlbaum Associates (1988).

[18] Gonzalo JD Haidet P Papp KK Wolpaw DR Moser E Wittenstein RD . Educating for the 21st-century health care system: an interdependent framework of basic, clinical, and systems sciences. Acad Med. (2017) 92:35–9. doi: 10.1097/ACM.0000000000000951 26488568

[19] Tavakol M Dennick R. Making sense of Cronbach's alpha. Int J Med Educ. (2011) 2:53–5. doi: 10.5116/ijme.4dfb.8dfd, 28029643 PMC4205511

[20] Wilson AB Brooks WS Edwards DN Deaver J Surd JA Pirlo OJ . Survey response rates in health sciences education research: a 10-year meta-analysis. Anat Sci Educ. (2024) 17:11–23. doi: 10.1002/ase.2345, 37850629

[21] McClintock AH Fainstad T Blau K Jauregui J. Psychological safety in medical education: a scoping review and synthesis of the literature. Med Teach. (2023) 45:1290–9. doi: 10.1080/0142159X.2023.2216863, 37266963

[22] Torralba KD Loo LK Byrne J. Does psychological safety impact the clinical learning environment for resident physicians? Results from the VA'S learners' perceptions survey. J Grad Med Educ. (2016) 8:699–707. doi: 10.4300/JGME-D-15-00719.1, 28018534 PMC5180524

[23] Almutairi A Alrebdi M Kalevaru CS Jahan S. Three years versus four years Saudi board family medicine program: graduates' academic performance, perceptions, burnout, and satisfaction with professional life. J Family Med Prim Care. (2023) 12:2786–96. doi: 10.4103/jfmpc.jfmpc_917_23, 38186826 PMC10771212

[24] Alshammari SA. Preparedness to implement “a family physician for every family,” which is the magic recipe for cost-effective health care for all: viewpoint. J Nat Sci Med. (2023) 6:95–100. doi: 10.4103/jnsm.jnsm_141_22

[25] Thistlethwaite JE Bartle E Chong AAL Dick ML King D Mahoney S . A review of longitudinal community and hospital placements in medical education: BEME guide no. 26. Med Teach. (2013) 35:e1340–64. doi: 10.3109/0142159X.2013.806981, 23848374

[26] Glynn LG O'Regan A Casey M Hayes P O'Callaghan M O'Dwyer P . Career destinations of graduates from a medical school with an 18-week longitudinal integrated clerkship in general practice. Ir J Med Sci. (2021) 190:185–191. doi: 10.1007/s11845-020-02260-0 32462491 PMC7846533

[27] Orsmond P McMillan H Zvauya R. It's how we practice that matters: professional identity formation and legitimate peripheral participation in medical students: a qualitative study. BMC Med Educ. (2022) 22:91. doi: 10.1186/s12909-022-03107-1 35139839 PMC8830078

[28] Dornan T Conn R Monaghan H Kearney G Gillespie H Bennett D. Experience based learning (ExBL): clinical teaching for the twenty-first century. Med Teach. (2019) 41:1098–105. doi: 10.1080/0142159X.2019.1630730, 31382787

[29] Taylor D Picker B Woolever D Thayer EK Carney PA Galper AB. A pilot study to address tolerance of uncertainty among family medicine residents. Fam Med. (2018) 50:531–8. doi: 10.22454/FamMed.2018.634768, 30005116

[30] Reis-Dennis S Gerrity MS Geller G. Tolerance for uncertainty and professional development: a normative analysis. J Gen Intern Med. (2021) 36:2408–13. doi: 10.1007/s11606-020-06538-y, 33532966 PMC7853704

[31] Hafiz AM Senturk E Teker C Sarikaya O. Factors affecting the level of reflective thinking and clinical decision-making skills in medical faculty students. Med Bull Sisli Etfal Hosp. (2023) 57:543–51. doi: 10.14744/SEMB.2023.52223, 38268663 PMC10805058

[32] Winkel AF Yingling S Jones AA Nicholson J. Reflection as a learning tool in graduate medical education: a systematic review. J Grad Med Educ. (2017) 9:430–9. doi: 10.4300/JGME-D-16-00500.1, 28824754 PMC5559236

[33] Cruess RL Cruess SR Boudreau JD Snell L Steinert Y. A schematic representation of the professional identity formation and socialization of medical students and residents: a guide for medical educators. Acad Med. (2015) 90:718–25. doi: 10.1097/ACM.0000000000000700, 25785682

[34] Sternszus R Steinert Y Razack S Boudreau JD Snell L Cruess RL. Being, becoming, and belonging: reconceptualizing professional identity formation in medicine. Front Med. (2024) 11:1438082. doi: 10.3389/fmed.2024.1438082, 39257893 PMC11383779

[35] Marmot M Friel S Bell R Houweling TAJ Taylor S Commission on Social Determinants of Health. Closing the gap in a generation: health equity through action on the social determinants of health. Lancet. (2008) 372:1661–9. doi: 10.1016/S0140-6736(08)61690-6, 18994664

[36] Gonzalo JD DeWaters AL Thompson B Mazotti L Riegels N Cooney R . System citizenship: re-envisioning the physician role as part of the sixth wave of professionalism. Am J Med. (2023) 136:596–603. doi: 10.1016/j.amjmed.2023.03.001, 36889491

[37] Khanna P Roberts C Lane AS. Designing health professional education curricula using systems thinking perspectives. BMC Med Educ. (2021) 21:20. doi: 10.1186/s12909-020-02442-5 33407403 PMC7789213

[38] Ericson A Bonuck K Green LA Conry C Martin JC Carney PA. Optimizing survey response rates in graduate medical education research studies. Fam Med. (2023) 55:304–10. doi: 10.22454/FamMed.2023.750371, 37310674 PMC10622096

[39] Kraft MA. Interpreting effect sizes of education interventions. Educ Res. (2020) 49:241–53. doi: 10.3102/0013189X20912798

[40] Soemantri D Herrera C Riquelme A. Measuring the educational environment in health professions studies: a systematic review. Med Teach. (2010) 32:947–52. doi: 10.3109/01421591003686229, 21090946

