# Supplementary material for: Systems thinking as the integrative competency in undergraduate Family Medicine education: a cross-sectional evaluation of an integrated primary care block
Source: Front Med (Lausanne). 2026 Jul 31;13:1870311. doi: 10.3389/fmed.2026.1870311 (PMC13474014; doi:10.3389/fmed.2026.1870311)
Supplement: Supplementary file 1 [file Supplementary_file_1.docx]

**Supplementary File 1. STROBE Statement Checklist**

*Checklist of items that should be included in reports of cross-sectional studies*

**Manuscript:** Systems Thinking as the Integrative Competency in Undergraduate Family Medicine Education: A Cross-Sectional Evaluation of an Integrated Primary Care Block.

| **Section / Topic** | **#** | **STROBE Recommendation** | **Page / Section** |
| --- | --- | --- | --- |
| **TITLE AND ABSTRACT** | | | |
| **Title and abstract** | 1(a) | Indicate the study's design with a commonly used term in the title or the abstract | Title; Abstract Methods |
|  | 1(b) | Provide in the abstract an informative and balanced summary of what was done and what was found | Abstract (structured) |
| **INTRODUCTION** | | | |
| **Background / rationale** | 2 | Explain the scientific background and rationale for the investigation being reported | Section 1 (Introduction) |
| **Objectives** | 3 | State specific objectives, including any prespecified hypotheses | Section 1 (Introduction), final paragraph |
| **METHODS** | | | |
| **Study design** | 4 | Present key elements of study design early in the paper | Section 2.1 |
| **Setting** | 5 | Describe the setting, locations, and relevant dates, including periods of recruitment, exposure, follow-up, and data collection | Section 2.2 |
| **Participants** | 6(a) | Cross-sectional study: Give the eligibility criteria, and the sources and methods of selection of participants | Section 2.7 |
|  | 6(b) | Not applicable for cross-sectional studies (matching) | N/A |
| **Variables** | 7 | Clearly define all outcomes, exposures, predictors, potential confounders, and effect modifiers. Give diagnostic criteria, if applicable | Sections 2.5, 2.6 |
| **Data sources / measurement** | 8 | For each variable of interest, give sources of data and details of methods of assessment (measurement). Describe comparability of assessment methods if there is more than one group | Sections 2.5, 2.6 |
| **Bias** | 9 | Describe any efforts to address potential sources of bias | Sections 2.7, 2.8; Section 4.9 (Limitations) |
| **Study size** | 10 | Explain how the study size was arrived at | Section 2.7 (full enrollment census) |
| **Quantitative variables** | 11 | Explain how quantitative variables were handled in the analyses. If applicable, describe which groupings were chosen and why | Sections 2.6, 2.9 |
| **Statistical methods** | 12(a) | Describe all statistical methods, including those used to control for confounding | Section 2.9 |
|  | 12(b) | Describe any methods used to examine subgroups and interactions | Section 2.9 (regression with six predictors) |
|  | 12(c) | Explain how missing data were addressed | Section 3.1 (no missing data) |
|  | 12(d) | Cross-sectional study: If applicable, describe analytical methods taking account of sampling strategy | Section 2.9 (full census) |
|  | 12(e) | Describe any sensitivity analyses | Section 2.9 (multicollinearity assessment via VIF; model assumption checks) |
| **RESULTS** | | | |
| **Participants** | 13(a) | Report numbers of individuals at each stage of the study | Section 3.1 (126 enrolled, 96 responded) |
|  | 13(b) | Give reasons for non-participation at each stage | Section 4.9 (Limitations) |
|  | 13(c) | Consider use of a flow diagram | Not applicable (single survey administration) |
| **Descriptive data** | 14(a) | Give characteristics of study participants and information on exposures and potential confounders | Section 3.1; Table 1 |
|  | 14(b) | Indicate number of participants with missing data for each variable of interest | Section 3.1 (no missing data) |
| **Outcome data** | 15 | Cross-sectional study: Report numbers of outcome events or summary measures | Sections 3.2, 3.3; Table 1 |
| **Main results** | 16(a) | Give unadjusted estimates and, if applicable, confounder-adjusted estimates and their precision (e.g., 95% confidence interval). Make clear which confounders were adjusted for and why they were included | Section 3.5; Table 3 (B, SE, beta, 95% CI, VIF) |
|  | 16(b) | Report category boundaries when continuous variables were categorized | Section 2.5 (Likert response categories) |
|  | 16(c) | If relevant, consider translating estimates of relative risk into absolute risk for a meaningful time period | Not applicable |
| **Other analyses** | 17 | Report other analyses done, e.g., analyses of subgroups and interactions, and sensitivity analyses | Section 3.4 (correlations); Section 3.5 (VIF check) |
| **DISCUSSION** | | | |
| **Key results** | 18 | Summarize key results with reference to study objectives | Section 4.1 |
| **Limitations** | 19 | Discuss limitations of the study, taking into account sources of potential bias or imprecision. Discuss both direction and magnitude of any potential bias | Section 4.9 |
| **Interpretation** | 20 | Give a cautious overall interpretation of results considering objectives, limitations, multiplicity of analyses, results from similar studies, and other relevant evidence | Sections 4.1 to 4.7 |
| **Generalizability** | 21 | Discuss the generalizability (external validity) of the study results | Section 4.9 (single-institution limitation) |
| **OTHER INFORMATION** | | | |
| **Funding** | 22 | Give the source of funding and the role of the funders for the present study and, if applicable, for the original study on which the present article is based | Funding statement |

*Reference:* von Elm E, Altman DG, Egger M, Pocock SJ, Gotzsche PC, Vandenbroucke JP; STROBE Initiative. The Strengthening the Reporting of Observational Studies in Epidemiology (STROBE) statement: guidelines for reporting observational studies. Lancet. (2007) 370(9596):1453-1457.
