# Supplementary material for: Systems thinking as the integrative competency in undergraduate Family Medicine education: a cross-sectional evaluation of an integrated primary care block
Source: Front Med (Lausanne). 2026 Jul 31;13:1870311. doi: 10.3389/fmed.2026.1870311 (PMC13474014; doi:10.3389/fmed.2026.1870311)
Supplement: Supplementary file 2 [file Table_1.DOCX]

**Supplementary File 2**

**Full survey instrument: COM353 Family Medicine and Primary Care block post-block evaluation questionnaire**

This file reproduces the complete 42-item Likert questionnaire used to evaluate the COM353 block, together with the single global rating item and the optional open-ended question. Items were adapted from the Dundee Ready Education Environment Measure (DREEM) and the Groningen Reflection Ability Scale (GRAS) and supplemented with block-specific items developed by the Department's curriculum committee, as described in Section 2.5 of the main manuscript. Unless otherwise stated, each item was rated on a five-point Likert scale: 1 = Strongly Disagree, 2 = Disagree, 3 = Neutral, 4 = Agree, 5 = Strongly Agree.

**Domain 1. Learning Environment and Teaching Quality (Items 1-7)**

**1.** The COM353 block promoted active learning rather than passive memorization.

**2.** The learning objectives of COM353 were clear and consistently addressed.

**3.** Teaching sessions were well organized and logically structured.

**4.** Faculty encouraged questions, discussion, and critical thinking.

**5.** I felt comfortable expressing uncertainty during teaching sessions.

**6.** The learning environment was respectful and supportive.

**7.** The balance between lectures, discussions, and experiential activities was appropriate.

**Domain 2. Family Medicine and PHC Competencies (Items 8-14)**

**8.** COM353 improved my understanding of the core principles of Family Medicine.

**9.** I better understand the role of Primary Health Care in the healthcare system.

**10.** The block enhanced my understanding of continuity of care.

**11.** I gained insight into comprehensive care across age groups and conditions.

**12.** I better understand the importance of preventive care and health promotion.

**13.** The block highlighted the role of family physicians as first-contact clinicians.

**14.** I developed a clearer understanding of the scope of practice of family physicians.

**Domain 3. Experiential Learning and PHC Visits (Items 15-20)**

**15.** The PHC visits were well integrated into the COM353 block.

**16.** PHC visits helped me apply theoretical knowledge to real clinical settings.

**17.** I observed patient-centered care during PHC visits.

**18.** PHC exposure improved my understanding of long-term patient management.

**19.** I observed how family physicians address multiple concerns in a single visit.

**20.** PHC visits helped me understand care beyond diagnosis and prescriptions.

**Domain 4. Clinical Courage and Uncertainty Tolerance (Items 21-26)**

**21.** COM353 helped me become more comfortable managing clinical uncertainty.

**22.** I developed confidence in engaging with patients despite incomplete information.

**23.** The block encouraged clinical courage, including speaking up when unsure.

**24.** I am more comfortable acknowledging limitations in my knowledge.

**25.** I learned to tolerate ambiguity in clinical decision-making.

**26.** COM353 emphasized thoughtful reasoning rather than defensive medicine.

**Domain 5. Reflection and Professional Identity Formation (Items 27-32)**

**27.** Reflective activities helped me critically analyze my clinical experiences.

**28.** Writing reflections helped me link experiences to professional values.

**29.** Reflection increased my awareness of my assumptions and biases.

**30.** Reflection helped me integrate ethical, emotional, and clinical perspectives.

**31.** I am more comfortable reflecting on challenging or uncomfortable encounters.

**32.** The reflective component contributed to my development as a future physician.

**Domain 6. Systems Thinking and Community Orientation (Items 33-37)**

**33.** COM353 improved my understanding of healthcare as a system.

**34.** I gained insight into referral pathways and coordination of care.

**35.** The block emphasized collaboration with other healthcare professionals.

**36.** I better understand the physician's role in community-oriented care.

**37.** The block highlighted issues of equity, access, and social determinants of health.

**Domain 7. Overall Impact and Readiness (Items 38-42)**

**38.** COM353 influenced how I view my future clinical practice.

**39.** The block prepared me for future clinical rotations.

**40.** COM353 increased my interest in Primary Care and/or Family Medicine.

**41.** The skills developed in COM353 are relevant regardless of specialty choice.

**42.** Overall, COM353 was a valuable and effective block.

**Global rating (Item 43)**

**43.** Overall, how would you rate the COM353 block? (Rated on a five-point scale: 1 = Poor, 2 = Fair, 3 = Good, 4 = Very Good, 5 = Excellent.)

**Open-ended question (Item 44)**

**44.** Which aspect of the Primary Health Care experience had the greatest impact on your learning, and why? (Free-text response, optional.)

***Note.*** *Items 1-7 were adapted in part from the DREEM; Items 27-32 drew on the GRAS. Items addressing Family Medicine and PHC competencies, experiential PHC learning, clinical courage and uncertainty tolerance, systems thinking, and overall readiness were developed specifically for this block, informed by the tripartite uncertainty framework and the Health Systems Science framework cited in the main manuscript.*
